# Sensitization to secretoglobin and lipocalins in a group of young children with risk of developing respiratory allergy

**DOI:** 10.1186/s12948-017-0061-8

**Published:** 2017-03-03

**Authors:** Mizuho Nagao, Magnus P. Borres, Mayumi Sugimoto, Carl Johan Petersson, Satoshi Nakayama, Yu Kuwabara, Sawako Masuda, Patrik Dykiel, Takao Fujisawa

**Affiliations:** 1 Allergy Center and Department of Clinical Research, Mie National Hospital, IDD, Tsu, Mie Japan; 2 Thermo Fisher Scientific, Uppsala, Sweden; 3 Department of Women’s and Children’s Health, Uppsala University, Uppsala, Sweden; 4 Thermo Fisher Scientific, Uppsala, Tokyo Japan; 5 Department of Pediatrics, Mie National Hospital, Tsu, Mie Japan; 6 Department of Otorhinolaryngology, Mie National Hospital, Tsu, Mie Japan

**Keywords:** Asthma, Allergy, Children, Molecular allergy diagnostics, Pet allergen components, Component resolved diagnosis, Food allergy, Secretoglobin, Lipocalin, Sensitization

## Abstract

**Background:**

Multiple sensitizations in early age have been reported to be a risk for development of asthma. This study evaluates the emergence and evolution of IgE to aeroallergens among a cohort of children with physician-diagnosed atopic dermatitis and/or showing food allergy symptoms and to examine the relation to asthma development.

**Methods:**

Three-hundred and four children (median age 13.4 months at entry) with food allergy symptoms and/or atopic dermatitis without asthma at inclusion were analysed for IgE antibodies against food-, indoor- and outdoor-allergens and pet allergen components and correlated to the individuals’ outcome on asthma inception.

**Results:**

At 2 years of follow-up, physician-diagnosed asthma was 19.7% (n = 49) and asthma diagnosed any time was 24% (n = 67). History of persistent cough and asthma of father, combination of milk- and wheat-allergy symptoms and dual sensitization to house dust mite and Japanese cedar were independent risk factors for asthma. Sensitization to dog was the most prevalent inhalant allergen at entry. Asthma children had a higher proportion of sensitization to dog, cat and horse allergens at entry compared with non-asthma children. Being sensitized to both food, house dust mite and pet allergens was strongly associated with asthma (p = 0.0006). Component resolved diagnosis for dog and cat allergens showed that IgE antibodies to Can f 1 and Fel d 1 was common even at very young age.

**Conclusions:**

Early sensitization to inhalant allergens increases the risk of developing asthma as well as having milk and wheat allergy symptoms. Sensitization to dog, was common at an early age despite dog ownership. Sensitization to secretoglobin and lipocalins and less to serum albumins explained the pet sensitization.

## Background

The combination of atopic dermatitis and food allergy in young children reflect a strong risk for the development of asthma-like disease [1]. Symptomatic food allergy is especially associated with asthma among children with multiple or severe allergies [2]. Approximately one half of children with moderate to severe atopic dermatitis will have clinically relevant IgE antibodies to food allergens [3]. As atopic children grow older, the majority of allergen-specific IgE antibodies are directed against inhalant sources [4]. Sensitization to multiple allergens along with high IgE antibodies levels are features of severe atopic dermatitis in childhood. The knowledge that children with atopic dermatitis are at risk of developing asthma is poorly understood in general among health care providers. This is partially due to the fact that there is a wide variability in asthma development, 10–25%, in the risk estimate in longitudinal studies. A better recognition of the children at highest risk of developing asthma among the group of individuals with atopic dermatitis and food allergy is therefore needed.

At present, the identification of a child at high risk might not be possible with certainty. Current research points to some indicators including family history, history of asthma and allergies, early and severe sensitization to some food antigens and to aeroallergens and early viral infection associated with wheeze and adverse environmental exposures [5].

An atopic history of early life seems to be one of the key factors to identify an individual’s risk of persistent asthma. Illi et al. reported a cumulative prevalence of atopic dermatitis in the first 2 years of life of 21.5% among a general population of children [6]. When associated with allergic sensitization, atopic dermatitis was a good predictor of asthma at school age: the risk was not seen with atopic dermatitis in the absence of sensitization. Sensitization to hen’s egg seems to convey the greatest risk.

Birth cohorts in Europe, USA and Australia show that early sensitization and severe sensitization are risk factors for persistence of asthma [7–9]. Little is known about the timing and pattern of sensitization to individual aeroallergen in relation to the development of asthma in children with atopic dermatitis and food allergy. Sensitisation to animal and dust mite allergens are each a risk factor for the development of asthma. There seems to be a higher probability of wheeze for cat versus mite at a given IgE value among preschool children [10]. In the same study, summing IgE levels for mite, cat and dog at age 3 strengthened the risk for wheeze at age 5. Wisniewski et al. were not able to verify that multisensitization increase the risk for asthma [11]. Stoltz et al. have examined specific patterns of allergic sensitization in early childhood in relation to the risk of developing asthma and rhinitis [12]. They found that at 1 year of age only IgE antibodies to cat and dog were significantly associated with having asthma at age 6. Konradsen et al. have in a recent review stated that the prevalence of allergy to furry animal has been increasing in later years and allergy to cats, dogs or both is considered a major risk factor for the development of asthma and rhinitis [13].

We hypothesised that early onset of sensitization to aeroallergen in children with atopic dermatitis and/or showing food allergy symptoms beginning within the first years of life is important in order to identify those at most risk of developing asthma.

The aim of this study was to evaluate the emergence and evolution of IgE antibodies to aeroallergens among a cohort of children with physician-diagnosed atopic dermatitis and/or showing food allergy symptoms and to examine the relation to asthma development during a 2-year follow up.

## Methods

This study was based on children who participated in the IRAM (Impact of Rhinitis on Atopic March; UMIN000004157) cohort. The study was a prospective five visit study during 2 years. Beside medical history, children were also recorded for atopic dermatitis, allergic rhinitis, parents’ allergy/asthma and smoking history and age, gender, height and weight. Inclusion criteria for this study were confirmed atopic dermatitis and/or suspicion of food allergy. Suspicion of food allergy was based on clinical history of food-induced symptoms and corresponding sensitization to the food. In uncertain cases oral food challenges were added to determine allergy to the food in question following the procedure for food allergy diagnosis in the EAACI and Japanese guidelines [14]. As oral food challenges were not done in all children, the term “food allergy symptom” will be used instead of “food allergy” throughout this study combining the groups of children with confirmed food allergy and of children with suspicion of food allergy, respectively. The diagnosis of atopic dermatitis was made by the study physicians based on the criteria by Hanifin and Rajka [15]. Blood samples were taken for specific IgE antibody determinations (Table 1). Physician diagnosis of asthma, (at each visit), was based on the Japanese Paediatric Guideline for the treatment and management of bronchial asthma [16]. A previously known diagnosis of asthma was criteria for exclusion.

**Table 1:** Number of patients and prevalence figures

Number of children (entry/1 year/2 years)	304/270/242		
*Physician diagnosed*	Number of children (%)		
Atopic dermatitis (at entry)	210 (71)		
*Having symptoms for*			
Food allergy (at entry)	259 (88)		
Egg allergy (at entry)	232 (79)		
Milk allergy (at entry)	128 (44)		
Wheat allergy (at entry)	75 (26)		
History of prolonged cough	62 (21)		
Patients by sex (missing # = 2)	Girls	Boys	
	106 (36)	193 (64)	
Bronchial asthma (after 2 year)	49 (20)		
Bronchial asthma (anytime during follow up)	67 (~24)		
Age in month (at entry)/(sdv)	13.4/(5.5)		
Pet ownership (at entry)	Dog: 28 (9)	Cat: 4 (1)	Dog and cat: 31 (10)
Pet ownership (any time)	Dog: 38 (13)	Cat: 11 (4)	Dog and cat: 42 (14)

	Sensitization		
	Prevalence and 95% CI %^a^ and total # tested (>0.35 kU_A_/L)		
	Entry (n = 304)	1 year (n = 270)	2 years (n = 242)
*Allergen extracts*			
m3 Aspergillus	3.6 (1.8–6.4)	4.4 (2.3–7.6)	9.9 (6.5–14.3)
m6 Alternaria	1.3 (0.4–3.3)	1.5 (0.4–3.7)	5.0 (2.6–8.5)
g3 Orchard grass	4.3 (2.3–7.2)	15.2 (11.1–20.0)	27.7 (22.2–33.8)
t24 Japanese cypress	3.0 (1.4–5.6)	12.6 (8.9–17.2)	32.8 (26.9–39.1)
f1 Egg white	86.2 (81.8–89.9)	83.7 (78.8–87.9)	85.1 (80.0–89.4)
f2 Milk	54.6 (29.5–40.5)	56.3 (50.2–62.3)	57.0 (50.5–63.3)
f4 Wheat	44.4 (38.7–50.2)	45.2 (39.1–51.3)	50.4 (43.9–56.9)
e1 Cat	18.8 (14.5–23.6)	25.2 (20.1–30.8)	33.5 (27.6–39.8)
e3 Horse d	6.3 (3.7–9.9)^a^	5.4 (3.0–9.0)^b^	7.4 (4.4–11.6)^c^
e5 Dog d	36.8 (31.4–42.5)	44.4 (38.4–50.6)	47.1 (40.7–53.6)
w1 Comm ragw	4.6 (2.6–7.6)	17.4 (13.1–22.5)	26.0 (20.6–32.0)
t17 Japanese cedar	3.9 (2.1–6.8)	24.4 (19.4–30.0)	51.7 (45.2–58.1)
d1 D.pteronyssinus	30.6 (25.5–36.1)	57.8 (51.6–63.7)	74.8 (68.8–80.1)

		Prevalence and 95% CI %^a^ and total # tested (>0.1 kUA/L)		
*Allergen components*				
Bos d 6	(Milk)	22.7 (18.1–27.8)	25.6 (20.5–31.2)	24.0 (18.7–29.9)
Can f 1	(Dog)	18.4 (14.2–23.2)	20.7 (16.1–26.1)	26.4 (21.0–32.5)
Can f 2		7,6 (4.9–11.1)	11.5 (7.9–15.9)	14.5 (10.3–19.5)
Can f 3		9.5 (6.5–13.4)	12.2 (8.6–16.7)	12.8 (8.9–17.7)
Can f 5		8.2 (5.4–11.9)	10.0 (6.7–14.2)	12.0 (8.2–16.8)
Fel d 1	(Cat)	13.2 (9.6–17.5)	16.3 (12.1–21.3)	25.2 (19.9–31.2)
Fel d 2		8.6 (5.7–12.3)	12.2 (8.6–16.7)	12.0 (8.2–16.8)
Fel d 4		9.9 (6.8–13.8)	9.3 (6.1–13.4)	15.7 (11.4–20.9)
Equ c 1	(Horse)	7.6 (4.9–11.1)^a^	8.5 (5.5–12.5)^b^	10.7 (7.2–15.3)^c^

^a^n = 271

^b^n = 257

^c^n = 230

The informed consent was signed by a legal guardian and the protocol was reviewed and approved by the ethics committee in Mie National Hospital, Japan.

Serum samples were analysed for IgE antibodies using the ImmunoCAP® system according to the manufacturer’s guidelines (Phadia AB, Uppsala, Sweden).

Statistical analyses were performed using the SAS® 9.3 (SAS Institute Inc., Cary, USA) and R 3.2.3 (R Foundation for Statistical Computing, Vienna, Austria,). All tests were two-sided and using significance level of 5%. The Chi square test or, when appropriate, Fischer’s exact test were used to compare proportions. The Mann–Whitney test was used to compare specific IgE levels at entry and after 2 year follow up. The effect of demographic variables, medical history and sensitisation to allergen components were jointly investigated using a logistic regression model.

## Results

Number of patients and prevalence figures are presented in Table 1. The average number of allergens that a child was sensitized against was at entry 3.0 (SD 2.4; range 0–13) and after 2 years, 5.1 (3.2; 0–13). Amongst the children that were diagnosed with bronchial asthma anytime during the course of the study (n = 67), the corresponding figure at entry was 3.8 (2.8; 0–13) whilst in the group of children (n = 236) that did not develop asthma the mean number of positive allergens was 2.8 (2.2; 0–13) (p = 0.019). Corresponding figures after 2 years were 6.2 (3.4; 0–13) and 4.7 (3.1; 0–13) respectively (p = 0.025). At entry, among the children diagnosed with asthma 6% were not sensitized to any of the tested allergens, 16% were monosensitized, 30% were sensitized to two up to a maximum of three allergens and 48% to more than three allergens. Corresponding numbers for the non-asthma group were 11, 20, 39 and 30% respectively.

Forty-nine out of the 67 (73%) children that were diagnosed with asthma anytime during the 24 months follow-up time period had an asthma diagnosis at the last visit.

Asthma were significantly associated with persistent cough (OR 3.7, 95% CI 1.75–7.80), asthma of father (2.39, 1.14–5.04) and milk allergy symptoms (2.48, 1.22–5.05), when using >0.1 kU_A_/L as cut-off. When using >0.34 kU_A_/L as cut-off, house dust mites in addition to the same factors as mentioned above were associated with higher risk of asthma diagnosis (Table 2).

**Table 2:** For each variable—likelihood of being diagnosed with asthma

Parameter	>0.1 kUA/L			>0.35 kUA/L		
	Odds ratio	95% CI	p value	Odds ratio	95% CI	p value
Asthma of father	2.39	1.14–5.04	*0.022*	2.19	1.04–4.63	*0.039*
Atopic dermatitis	1.32	0.64–2.73	0.459	1.23	0.59–2.56	0.581
Cat dander sensitivity	0.64	0.27–1.51	0.307	1.11	0.46–2.67	0.813
Dog dander sensitivity	1.00	0.41–2.43	0.995	0.79	0.35–1.78	0.573
Egg allergy symptoms	0.79	0.34–1.85	0.586	0.80	0.34–1.86	0.604
HDM sensitivity	1.81	0.84–3.93	0.131	2.45	1.15–5.22	*0.021*
Hist. of pneum./bronch.	2.06	0.57–7.43	0.267	1.91	0.53–6.96	0.324
Horse dander sensitivity	1.11	0.41–3.00	0.833	0.55	0.14–2.19	0.392
Milk allergy symptoms	2.48	1.22–5.05	*0.013*	2.52	1.26–5.05	*0.009*
Persistent cough	3.69	1.75–7.80	*0.001*	3.66	1.73–7.76	*0.001*
Smoking in family	1.04	0.53–2.04	0.915	1.04	0.53–2.07	0.900
T17 sensitivity	1.88	0.74–4.76	0.184	2.22	0.53–9.31	0.276
Wheat allergy sympt.	1.60	0.76–3.37	0.215	1.70	0.81–3.55	0.158
Milk wheat al. sympt	3.97	1.47–10.68	*0.003*	4.29	1.60–11.47	*0.002*
HDM and t17 sens.	3.41	1.16– 9.96	*0.013*	5.43	1.18–24.96	*0.015*

Odds ratio with 95% confidence interval and p value. Shown for cut-off s: ≥0.1 and ≥0.35 kUA/L respectively

Italic values are statictically significant (p < 0.05)

Having a combination of milk and wheat allergy symptoms and house dust mite and cedar pollen sensitization had a likelihood of 38 and 45% respectively of being diagnosed with asthma anytime during follow-up (Table 3). Children with wheat allergic symptoms did not differ in their sensitization pattern at entry or at 2 year follow up compared to children with no wheat allergy symptoms. The results from the logistic regression were in line with this finding as sensitisation to Japanese cedar and wheat allergy symptoms were associated with increased probability of asthma diagnosis during follow-up, although they did not reach statistical significance (Table 2).

**Table 3:** Distribution of patients diagnosed with asthma in groups of patients with (A) milk and wheat allergy symptoms (yes or no) and (B) in groups of patients sensitized or not sensitized to house mite and cedar pollen (yes or no)

Symptoms of			
Milk allergy	Wheat allergy	Number of patients: asthma/total	% Diagnosed with asthma
A			
N	N	24/137	17.5
N	Y	5/28	17.9
Y	N	19/81	23.5
Y	Y	18/47	38.3

H. dust mite	Cedar	Number of patients: asthma/total	% Diagnosed with asthma
B			
N	N	36/210	17.1
N	Y	1/1	(100.0)
Y	N	25/82	30.5
Y	Y	5/11	45.5

**Fig. 1:**
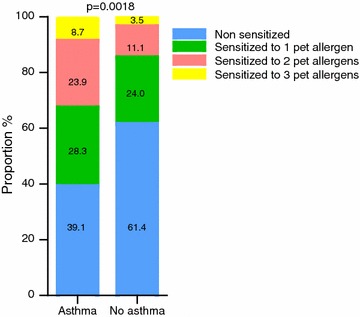
Proportional distribution of allergen sensitizations at study entry to cat and/or dog and/or horse in patients with and without current asthma

Children diagnosed with asthma at follow up were significantly more likely to be multi-sensitized to animals compared to the non-asthmatic children at entry (Fig. 1). Thirty-three percent of the asthma children were sensitized to two or more animals compared to 15% of the non-asthmatic children.

**Fig. 2:**
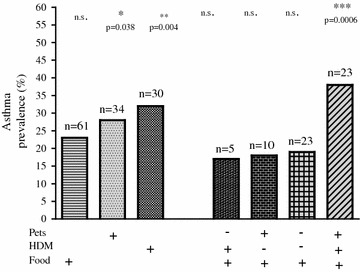
Asthma prevalence in relation to sensitization to pets, HDM and food allergens. p Values were calculated when compared with the group without any sensitization, *n.s.* not significant. Not shown: Pets(+)HDM(−)Food(−), Pets(−)HDM(+)Food(−) and Pets(+)HDM(+)Food(−) because of too few number of observations in these combinations

Sensitization to three different groups of allergens, pets, house dust mite and food, was analysed one by one or in combination in respect to asthma prevalence. Being sensitized to pet allergens and to house dust mite allergen independently of each other was associated with asthma (Fig. 2). Sensitization to all three groups in their combination was strongly associated with asthma (p = 0.0006).

Two hundred and ten children had atopic dermatitis at entry and 35 children were diagnosed with asthma at 2 years follow up. Totally 48 children were diagnosed with asthma anytime during the study. Mean number of positive allergens among these were 6.3 and 5.7 respectively. Corresponding figures in the non-asthmatic groups were 5.0 and 4.0 respectively. An increased risk of developing asthma (odds ratio = 1.22, CI 1.03–1.45, p = 0.02) was observed with increased number of positive allergens amongst the AD children.

**Fig. 3:**
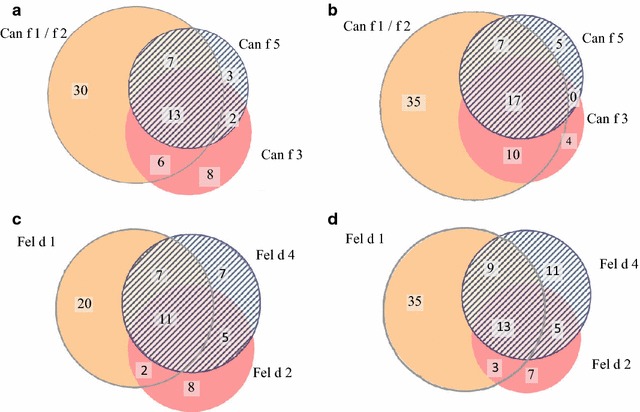
Proportional Venn diagram of the number of children sensitized to the allergen components from dog (**a**, **b**) and cat (**c**, **d**) at entry and after 2 years (cut-off = 0.1 kUA/L)

Ninety-six percent (189/196) of the dog sensitized individuals had complete data set for measurements of IgE to Can f 1, Can f 2, Can f 3 and Can f 5 at entry. Overall sensitization to least one of these components was 37% (n = 69). Sensitization was highest for the lipocalins Can f 1/Can f 2 (30%, n = 56) followed by Can f 3 (15%, n = 29) and Can f 5 (13%, n = 25) (Fig. 3a). Fifteen percent were sensitized to at least two dog components whilst 7% were sensitized to all three components. Corresponding figures at 2 year follow up from 151 children with complete data set showed an overall sensitization to at least one component was 52% (n = 78). The most prevalent sensitization was found for Can f 1/Can f 2 (46%, n = 69) followed by Can f 3 (21%, n = 31) and Can f 5 (19%, n = 29) (Fig. 3b). At 2 year follow up, 23% were sensitized to at least two dog components whilst 11% were sensitized to all three components. Thirty-five (71%) of the asthma children were dog sensitized at entry. All these 35 children had complete data set for the dog components and 23 (47%) of them were positive to at least one of these components. In the group of non-asthmatic children after 2 years followed up 119 children were dog sensitized (62%) and 115 of them had complete data set. Fifty (29%) children were positive to at least one of the dog components.

Ninety-four percent (102/108) of the cat sensitized children had complete data set for cat components Fel d 1, Fel d 2 and Fel d 4 (cut-off = 0.1 kU_A_/L). Overall sensitization to at least one of these components was 59% (n = 69). Sensitization to Fel d 1 was found to be most prevalent (39%, n = 30) followed by Fel d 4 (29%, n = 26) and Fel d 2 (25%, n = 26) (Fig. 3c). Twenty-five percent were sensitized to at least two cat components whilst 11% were sensitized to all three components. Corresponding figures after 2 years follow up from 115 children with complete data set showed an overall sensitization to at least one of the components of 72%. Sensitization to Fel d 1 was most prevalent (52%, n = 60) followed by Fel d 4 (33%, n = 38) and Fel d 2 (24%, n = 28) (Fig. 3d). Here, 26% were sensitized to at least two cat components whilst 11% were sensitized to all three components. In the group of the 49 children that eventually were diagnosed with asthma 31 (63%) were cat sensitized. All these 31 children had complete data set and 19 (61%) were found to be positive to at least one of the components. Forty-three percent (84/193) of the non-asthmatic children were cat sensitized after 2 years followed up and they all had complete data set. In this group 64 (76%) of the children were positive to at least one of these components.

## Discussion

In this study we examined predictors of asthma among children with atopic dermatitis and/or food allergy symptoms. We found that being sensitized to both house dust mite and cedar pollen or having both milk and wheat allergy symptoms were associated with asthma development during a 2 year follow up. A high proportion were sensitized to dogs and cats and mainly due to sensitization to secretoglobin and lipocalins and less due to serum albumins.

Sensitization to cat and dog components per se were not better predictors of asthma development than sensitization to cat and dog whole allergens. However, Wisniewski et al. found that both Fel d 1 and Fel d 4 were identified as predictors of wheeze among cat sensitized children with atopic dermatitis [11]. One explanation to the different findings could be differences in age in the two study population. Median age in the American study was 7.5 years compared to the median age of 1.5 year in our study [11]. This means that the asthma diagnosis will be based on a much shorter observational period compared to the study of Wisniewski et al.

Having milk and wheat allergy symptoms in combination was associated with increased risk of asthma diagnosis during the follow up. Eighty-eight percent of the included children had food allergy symptoms and 47 of them had this milk- and wheat allergy symptoms combination, of which 40% developed asthma within 2 years. In comparison, out of the children with a single milk or wheat allergy symptom 23 and 18% developed asthma respectively. We know from the literature that especially egg but also milk sensitization are associated with development of IgE to respiratory allergens [17–19]. Few prospective studies have studied the impact of wheat allergy/sensitization on asthma development. Illi et al. showed that early atopic sensitization played a major role for the prognosis of atopic dermatitis and sensitization to wheat showed the strongest association. The group with early atopic dermatitis and wheeze showed sensitization to wheat, cat, mite, soy and birch. Nilsson et al. found that 72% of children with a challenge verified wheat allergy had or have had milk allergy and 75% reported asthma symptoms pointing to a relationship between milk- and wheat-allergy with asthma [20]. The reason why symptoms for egg allergy did not single out as a risk factor for asthma in our study is probably due to that most of the children included in the study had egg allergic symptoms at entry. We observed in our study that sensitization to HDM, pets and food was higher in children with asthma compared with the non-asthmatics. Furthermore we could see that there was a higher prevalence of sensitization to these allergen groups in children with AD than in the non-AD children (79 vs 21%, data not shown). Wisniewski et al. [11] also reported that the prevalence of sensitization to these allergens increased considerable in AD-children less than 2 years of age up to 15 years of age. Even though the follow up period in our study period was limited to 24 months, from 1 year of age to the age of around 3 years we could also observed a clear increase in the sensitization prevalence for many of these allergens. However, the short follow up period and the young age of the children in our study limits the possibility to compare our study results with other longitudinal studies with long follow up periods.

Typical symptom patterns are important for the establishment of an asthma diagnosis. These include recurrent episodes of cough, wheeze, difficulty in breathing, chest tightness, and respiratory infections [21]. We found in our population that persistent cough was strongly associated with development of asthma. Cough is the most common cause for new visits in childhood ambulatory care and it is important to remember that this symptom is not pathognomonic for asthma and may occur as a result of several different conditions. Ongoing attempts are being made to simplify prediction tools for identifying children with wheeze or cough who are at risk for asthma. Pescatore et al. provided a simple, low-cost and non-invasive questionnaire based method to predict the risk of later asthma in symptomatic preschool children [22]. However, we do need objective biomarkers for diagnosing asthma in young children as cough and other symptoms may occur as a result of several different respiratory conditions. Being sensitized to both house dust mite and cedar pollen increased the risk significantly of developing asthma during the follow up period. A limitation of the study is that this finding is based on only eleven children sensitized at this early age and this finding has to be verified by others.

The high sensitization prevalence for dog was a surprise and no equivalent data has been found in the literature. This is surprisingly high as only 19% of the children were exposed to dog at home. One possible explanation is cross-reactions between bovine and pet albumins. However, Bos d 6 was positive in 23% of the children but Fel d 2 and Can f 3 were only positive in 9 and 8% respectively. This explanation could be partially true as we could document that monosensitisation to pet albumins decreased significantly over the study period, which might be an effect of development of milk tolerance. Instead the most likely explanation is that the majority of the animal sensitized individuals are genuinely sensitized to the secretoglobin (Fel d 1) and lipocalin (Can f 1). Eighteen percent were positive to the major dog component Can f 1 and 13% to Fel d 1, the major cat component already at entry. We have no similar data to compare this with but the picture looks very different from pet component pattern in older children. Bjerg et al. performed a population-based study of animal component sensitization, asthma and rhinitis in schoolchildren and found that 32% were sensitized to dog and 30% to cat [23]. Furthermore, only 5 and 4 of the children that developed asthma were sensitized to Can f 3 and Fel d 2 respectively, which gives a total different sensitization pattern to animal components. Simpson et al. have described patterns of IgE responses to multiple allergen components using latent variable modelling in association with different clinical symptoms [24]. They found that sensitization to the group with component from domestic pets was strongly associated with asthma. Uriatre and Sastre were able to associate IgE sensitization to the different animal molecules with asthma severity [25]. We could not verify this finding but found that if sensitized to two or more animals at an early age, you are more likely to develop asthma. This seems to be even more likely if you are also sensitized to house dust mite and food on the same time. However, our observation period was only 2 years, which is a drawback if studying asthma development in childhood.

## Conclusions

We conclude that early sensitization to inhalant allergens increase the risk of developing asthma as well as having milk and wheat allergy symptoms. The most common inhalant allergens causing sensitisation was dog allergen, but prevalence and concentration remained stable over the 2 year follow up compared to house dust mite and cedar pollen. The dog and cat sensitisation was mainly explained by sensitisation to major allergens and not cross reactions to serum albumin.

## Abbreviations

Specific IgE: specific immunoglobulin E
SD: standard deviation
OR: odds ratios
kUA/L: kilo units (arbitrary) per litre
AD: atopic dermatitis
